# *N*- and *O*-glycan cell surface protein modifications associated with cellular senescence and human aging

**DOI:** 10.1186/s13578-016-0079-5

**Published:** 2016-02-18

**Authors:** Yoko Itakura, Norihiko Sasaki, Daisuke Kami, Satoshi Gojo, Akihiro Umezawa, Masashi Toyoda

**Affiliations:** 1 Research Team for Geriatric Medicine (Vascular Medicine), Tokyo Metropolitan Institute of Gerontology, 35-2 Sakae-cho, Itabashi-ku, Tokyo, 173-0015 Japan; 2 Department of Regenerative Medicine, Kyoto Prefectural University of Medicine, 465 Kajii-cho, Kawaramachi-Hirokoji, Kamigyo-ku, Kyoto, 602-8566 Japan; 3 Department of Reproductive Biology, National Research Institute for Child Health and Development, 2-10-1 Okura, Setagaya-ku, Tokyo, 157-8535 Japan

**Keywords:** Cellular senescence, Human aging, Glycan, Lectin microarray

## Abstract

**Background:**

Glycans play essential roles in biological functions such as differentiation and cancer. Recently, glycans have been considered as biomarkers for physiological aging. However, details regarding the specific glycans involved are limited. Here, we investigated cellular senescence- and human aging-dependent glycan changes in human diploid fibroblasts derived from differently aged skin donors using a lectin microarray.

**Results:**

We found that α2-6sialylated glycans in particular differed between elderly- and fetus-derived cells at early passage. However, both cell types exhibited sequentially decreasing α2-3sialylated *O*-glycan structures during the cellular senescence process and showed similar overall glycan profiles.

**Conclusions:**

We observed a senescence-associated decrease in sialylation and increase in galactose exposure. Therefore, glycan profiling using lectin microarrays might be useful for the characterization of biomarkers of aging.

**Electronic supplementary material:**

The online version of this article (doi:10.1186/s13578-016-0079-5) contains supplementary material, which is available to authorized users.

## Background

The cell surface is covered with various glycoproteins, which play crucial roles in biological functions such as cell–cell adhesion, maintenance of protein structure, and molecular recognition. Dynamic changes in cell surface glycosylation regulate cellular function during development, differentiation, and survival. Recently, glycans have been considered as biomarkers for physiological aging. Certain *N*-glycans of IgG and α1-antitrypsin have been associated with chronological age and the physiological parameters of inflammation or cardiovascular disease [1, 2]. In addition, *N*-glycan alteration associated with age and gender has been reported [3]. However, details of the changes of specific glycans including both *N*- and *O*-glycan forms on glycoproteins upon cellular senescence and their biological functions are unclear. Therefore, investigation of the cell surface glycan changes during the senescence process will be helpful to better understand their biological function in human aging.

Various human diploid fibroblasts have been used as model systems of cellular senescence. A series of human diploid fibroblasts (TIGs) have been well characterized with respect to morphological alteration, chromosome constitution, cellular life span, telomere attrition and length, cellular protein content, and glycosylation [4–8]. In addition, changes in the cell surface glycans of several of these lines during the senescence process have been analyzed using lectin, demonstrating a decrease of α2-6sialylation of *N*-glycan in senescent TIG-3 lung fibroblasts in vitro [9]. Furthermore, it has been suggested that the cell surface sialic acid level in senescent WI-38 human fetal lung diploid fibroblasts is low, and a great amount of sialic acid is transferred to asialo acceptors in the absence of exogenous acceptors as measured by a sialyltransferase assay [10]. The change of cell surface glycan composition during cellular senescence has also been demonstrated on the basis of lectin affinity in the human fetal lung fibroblast lines HSC172 and IMR-90 [11, 12]. Further, it has been suggested that the surface glycans of IMR-90 control both cell growth and function because they were observed to change prior to morphological alteration [13]. Based on these reports, it appears that various glycan changes on lung fibroblasts are associated with aging. However, these data reflected only partial analysis of glycosylation, examining alternately *N*- and *O*-glycan, and selected stepwise-aged cells. In order to analyze the glycome of cells, lectin microarrays have been developed [14, 15]. These arrays represent an emerging technology that can be applied to the ultrasensitive detection of multiplex lectin-glycan interactions [16–18]; such glycan profiles, for example, have been used to distinguish the developmental stage and differentiation of various cells [19–23].

In this study, we compared consecutive cell surface glycan profiles of three human skin diploid fibroblast lines (the fetus-derived fibroblasts TIG-3S and the elderly-derived fibroblast lines TIG-101 and TIG-102), during extended cell culture. In addition, we identified specific glycan profiles associated with cellular senescence and human aging. Clarification of the senescence-dependent glycan profile specific to each derived cell type will contribute to a better understanding of the aging process of the skin at the cellular level.

## Results

### Cell growth rate and morphological change of fetus-derived TIG-3S cells

**Fig. 1:**
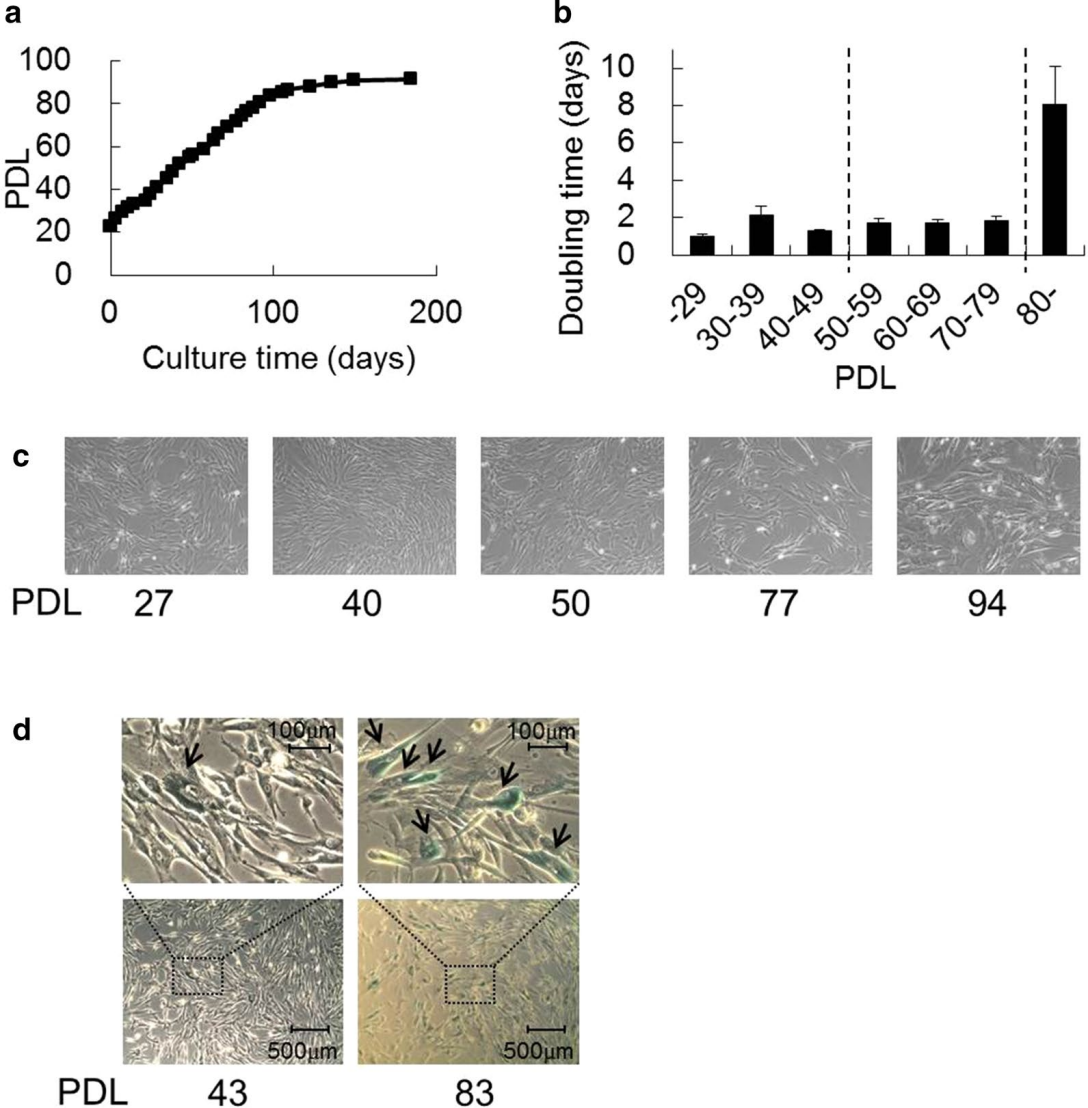
Growth curve and morphology of human diploid fetus-derived fibroblasts (TIG-3S). **a** Average proliferation of TIG-3S cells plotted as PDL for approximately 180-day culture (n = 3). **b** *Bar graph* representation of the average doubling time of the cells at each PDL (mean + SE, n = 4–22). **c** Cell shapes are shown at PDLs 27, 40, 50, 77, and 94. **d** TIG-3S cells at PDLs 43 and 83 were stained with SA-β-galactosidase. The* upper panels* are a magnification of the squared area in the* lower panels*. The *arrows* indicate stained cells

To observe the cell lifespan and growth rate of the TIG-3S line, we investigated cellular proliferation under stable conditions. Figure 1a shows the growth curve for TIG-3S (n = 3). Growth arrest was observed in the cells over population doubling level (PDL) 80 for 100-day culture. The doubling time of the TIG-3S line at early passages (PDL 27–50) was 1–2 days (Fig. 1b). At 80 % confluence, the cells exhibited an elongated shape (Fig. 1c, PDL 27, 40, and 50). Conversely, at late passages (>PDL 80), confluence was reached after a few weeks. The average doubling time after PDL 80 (n = 22) became approximately eight times as long as that observed during initial culture (Fig. 1b). At late passage, the cells appeared flat and expanded (Fig. 1c, PDL 94). In addition, they exhibited more senescence-associated (SA-)β-galactosidase activity than did early-passage cells, indicating that cellular senescence was induced (Fig. 1d).

### Cellular senescence-dependent changes of cell surface glycans in TIG-3S cells

**Fig. 2:**
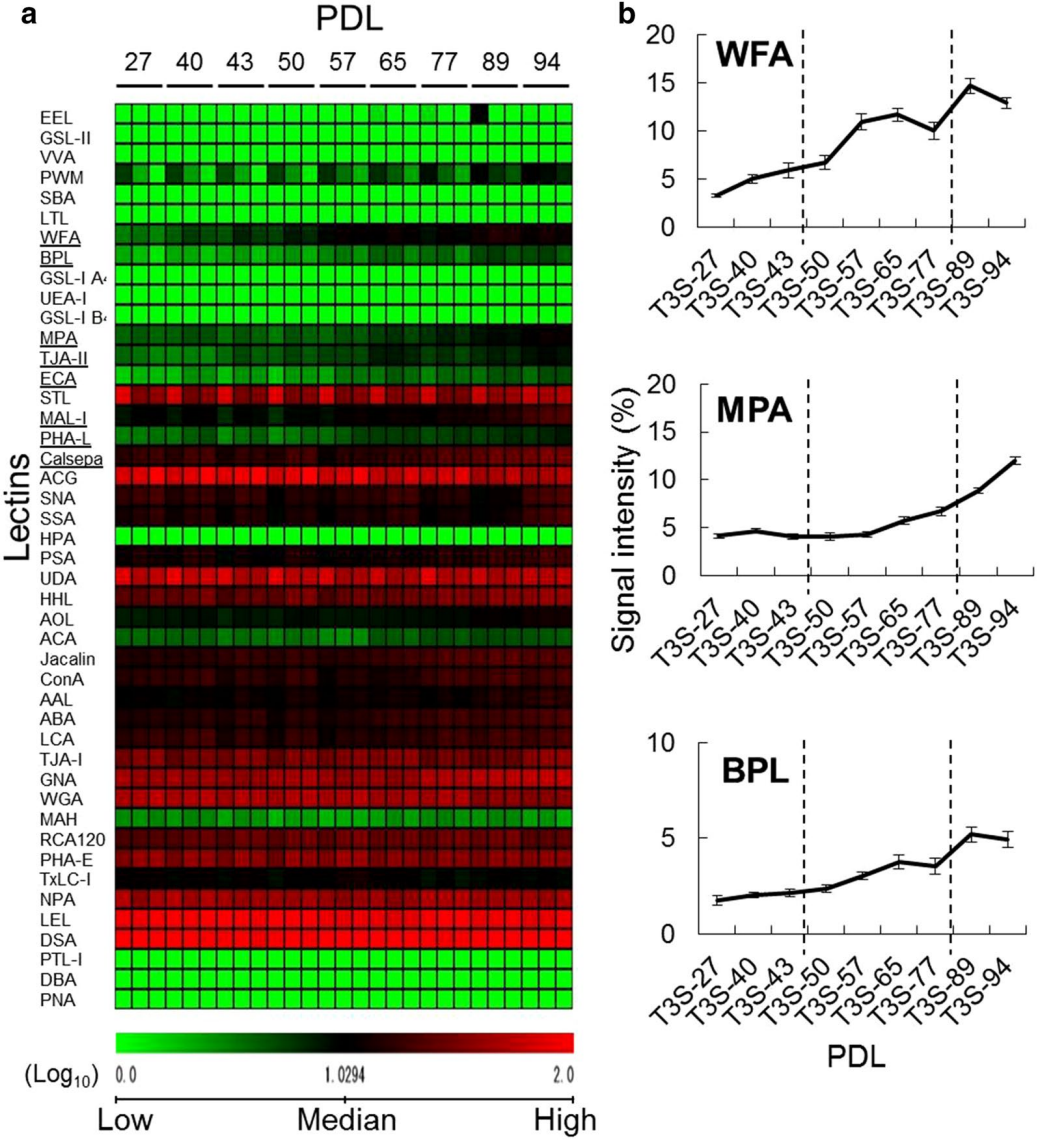
Lectin microarray analysis of cellular senescence in TIG-3S fibroblasts at various PDLs. **a** Heat map representation of the (log_10_-transformed) lectin microarray data from TIG-3S fibroblasts to compare the overall glycan profiles of the cells at different PDLs. The *rows* represent the lectins and the *columns* represent the PDLs (27–94). The *color scale* indicates low (*green*) to high (*red*) signal intensity. **b** *Line graph* representation of the signal intensity (%) at each PDL for changed lectins. There are three representative patterns including WFA, MPA and BPL. The data are represented as the mean ± SE (n = 3)

To investigate the glycan profiles associated with each PDL in TIG-3S fibroblasts, lectin microarray analysis was performed (Table 1). Figure 2a shows the heat map of TIG-3S lectin microarray signals, indicating that the signal intensities of some lectins significantly increased with passage. The signal intensity of WFA (Galβ1-3GalNAc- and GalNAcβ1-4GlcNAc-binder) gradually increased during cellular senescence process (Fig. 2b; Table 1). WFA is well known as a binder recognizing *O*-glycan. Signal intensities of three lectins, MPA (Galβ1-3GalNAc-binder), MAL-I (Siaα2-3Galβ1-4GlcNAc-binder), and Calsepa (High-Man- and Glc-binder) rapidly increased in late-passage cells (Fig. 2b; Additional file 1: Figure S1). Signal intensities of four other lectins, BPL (Galβ1-3GlcNAc-binder), TJA-II (Fucα1-2Galβ-binder), ECA (Galβ1-4GlcNAc-binder), and PHA-L (tri- and tetra-antennary complex type *N*-glycan-binder) slightly but significantly increased from middle passage (>PDL 50); these recognized *N*-glycan (Fig. 2b; Additional file 1: Figure S1). As the affinities of ECA and PHA-L increase with the branching number, the enhanced signals of ECA and PHA-L at middle and of MAL-I at late passages suggested that the large-antennary *N*-glycan increased, followed by a slight increase of the α2-3sialilated *N*-glycan form during cellular senescence process. Furthermore, the elevated WFA and MPA signals suggest that *O*-glycans such as the Galβ1-3GalNAc structure on the cell surface increased during cellular senescence as well.

**Table 1:** Lectin microarray data of TIG-3S, TIG-101, and TIG-102

Lectin/PDL	TIG-3S (%)									TIG-101 (%)					TIG-102 (%)				
	27	40	43	50	57	65	77	89	94	40	41	43	46	51	40	43	47	49	52
LTL	0.2	0.1	0.1	0.2	0.2	0.3	0.2	0.3	0.2	0	0.1	0	0.1	0.1	0.2	0.2	0.1	1.4	0.1
PSA	13.7	14.4	11.3	13.5	11.5	13.1	14.8	17.5	19.6	29.4	24.5	18.8	20.4	24.9	32.1	26.6	14.7	22.0	21.6
LCA	17.6	19.9	16.8	16.9	14.7	16.3	19.4	20.4	21.3	26.0	26.0	20.9	22.3	22.9	31.2	29.7	15.8	23.5	20.2
UEA-I	0.1	0.1	0.1	0.1	0	0.3	0.1	0.2	0.1	0	0.1	0	0.1	0	0.1	0.2	0.1	0.5	0
AOL	8.0	8.8	8.0	8.6	8.3	8.8	9.2	11.7	13.6	11.2	17.8	11.5	15.9	11.7	12.5	18.7	9.0	14.2	8.3
AAL	10.9	11.2	12.4	12.8	12.6	12.9	11.7	14.8	16.9	23.3	19.8	14.6	18.1	20.2	20.8	17.7	10.9	14.9	15.0
MAL-I	9.7	10.1	8.8	8.6	11.0	11.9	12.6	15.3	22.5	25.7	19.6	12.5	17.9	19.0	26.9	21.1	12.5	18.6	18.0
SNA	19.7	17.7	17.7	14.5	16.0	19.3	15.2	11.4	22.0	10.0	8.9	14.4	10.4	13.2	10.5	11.3	8.3	11.0	11.8
SSA	15.6	15.4	14.5	13.2	13.7	16.1	12.8	12.1	21.5	9.9	8.1	12.0	9.2	12.0	11.8	11.6	8.0	11.1	12.5
TJA-I	35.6	30.5	34.2	32.6	33.4	36.3	29.6	26.4	39.3	24.7	20.3	30.4	23.3	28.7	24.6	25.8	21.4	25.7	28.2
PHA-L	3.4	4.2	3.6	3.4	4.5	5.8	5.5	6.2	7.2	11.2	5.7	4.2	5.3	8.8	12.4	7.4	4.2	6.8	8.4
ECA	1.8	2.4	2.4	2.3	3.1	3.9	3.6	4.3	4.5	9.7	6.4	5.3	5.8	7.0	11.2	8.5	4.8	8.0	8.0
RCA120	22.9	26.3	28.0	28.6	29.3	27.8	29.6	29.9	25.5	25.9	27.9	28.5	26.0	27.0	28.6	29.1	26.0	26.6	27.8
PHA-E	40.2	38.3	38.3	36.9	41.1	35.4	36.5	37.0	37.5	36.5	38.3	36.9	38.1	38.2	38.9	37.4	31.2	34.1	29.8
DSA	100	99.7	94.7	95.9	100	100	96.7	100	100	100	100	97.5	100	100	100	100	100	100	100
GSL-II	0.3	0.2	0.2	0.1	0.2	0.4	0.4	0.4	0.3	0.5	0.5	0.1	0.4	0.2	0.4	0.5	0.2	0.4	0.3
NPA	46.7	50.2	44.7	45.6	40.6	41.5	52.1	44.7	45.6	49.7	57.8	59.7	52.5	50.7	58.1	58.0	45.1	49.4	46.0
ConA	16.4	18.3	16.4	17.7	14.4	14.4	20.3	20.8	20.7	27.3	34.4	30.7	32.9	25.0	32.0	39.3	26.6	32.1	24.1
GNA	44.4	44.8	39.7	47.7	40.8	39.7	51.5	56.4	60.1	40.2	45.8	41.5	40.8	44.0	49.5	45.6	30.5	40.4	38.1
HHL	26.6	29.6	25.3	30.8	26.6	27.8	33.3	35.8	40.9	43.9	49.3	46.1	39.9	45.8	50.0	47.5	33.1	42.9	41.8
ACG	81.3	81.8	88.5	83.3	77.2	63.4	71.0	51.6	49.9	39.1	51.9	59.4	48.8	47.5	45.1	50.9	56.3	47.5	47.9
TxLC-I	11.8	11.2	11.0	10.1	12.6	11.4	9.1	10.2	9.9	18.4	17.0	15.4	15.9	15.0	17.0	15.2	9.5	11.0	8.8
BPL	1.8	2.0	2.1	2.4	3.0	3.8	3.5	5.2	4.9	6.8	4.1	3.7	3.8	5.6	6.5	4.2	2.7	5.2	5.6
TJA-II	3.5	3.0	4.4	4.7	5.3	7.6	5.7	7.0	8.8	8.4	6.2	6.3	5.5	7.5	9.2	7.2	5.6	7.6	9.1
EEL	0.4	0.2	0.3	0.2	0.2	0.6	0.7	0.5	0.5	0.7	0.4	0.8	0.5	0.3	0.6	0.4	0.3	0.4	0.3
ABA	15.0	15.9	16.7	15.3	14.8	18.1	20.2	19.7	22.3	22.5	20.4	17.0	19.3	18.2	26.5	23.3	15.7	19.7	17.9
LEL	91.5	96.3	94.6	97.5	85.9	82.2	98.2	90.1	87.6	86.8	92.3	95.2	86.3	77.5	82.4	86.9	83.7	74.3	73.5
STL	50.8	45.6	46.8	48.1	47.1	46.0	43.0	48.2	54.9	66.2	60.6	58.2	56.3	57.1	56.5	55.2	54.3	52.9	53.0
UDA	63.5	70.1	57.4	64.5	56.4	52.3	60.5	54.9	57.0	56.9	69.3	76.0	65.6	64.9	64.4	67.6	70.1	66.5	64.0
PWM	2.9	3.2	2.7	3.1	4.0	4.4	5.0	6.9	9.2	15.8	13.7	14.1	11.7	13.6	14.5	12.3	8.7	11.2	11.5
Jacalin	15.3	15.9	18.1	18.0	16.5	19.5	22.7	24.3	23.3	28.1	26.8	22.7	26.1	24.5	29.4	28.3	19.5	23.1	21.8
PNA	0	0	0	0	0	0	0	0	0.1	0	0	0	0	0	0.1	0.1	0	0.1	0
WFA	3.3	5.0	5.9	6.7	10.9	11.7	10.0	14.7	12.9	13.9	8.6	9.0	8.5	14.2	14.9	10.4	8.1	12.1	16.5
ACA	3.9	4.3	3.6	3.5	2.9	4.4	4.8	5.1	4.5	5.9	4.1	2.7	4.2	3.6	6.5	5.0	2.7	4.2	3.1
MPA	4.1	4.7	4.1	4.0	4.3	5.7	6.7	8.9	12.0	16.0	11.7	7.7	9.8	11.2	14.6	10.7	6.1	9.1	9.4
HPA	0.1	0	0	0	0	0	0	0.1	0	0.3	0.1	0	0	0	0.2	0.2	0.1	0.1	0
VVA	0.2	0.1	0.1	0.1	0.2	0.4	0.4	0.4	0.5	0.7	0.2	0	0.1	0.2	0.5	0.1	0	0.1	0.2
DBA	0	0	0	0	0	0	0.1	0	0	0	0	0	0	0	0.1	0.1	0	0.4	0
SBA	0.1	0.1	0.1	0	0.1	0.3	0.2	0.2	0.3	0.6	0.2	0	0	0.2	0.6	0.3	0.1	0.2	0.7
Calsepa	18.1	19.4	16.3	19.2	15.0	17.5	22.5	27.2	31.4	36.2	35.9	31.7	32.4	34.4	40.4	38.5	23.9	29.8	28.6
PTL-I	0	0	0	0	0	0	0	0	0	0	0	0	0	0	0	0	0	0	0
MAH	2.9	2.9	2.6	2.3	2.1	2.6	2.9	2.9	2.4	9.7	5.9	4.2	6.5	4.5	9.8	7.7	5.3	6.5	3.8
WGA	53.6	52.9	50.2	50.3	50.9	45.9	49.7	37.5	36.9	30.9	36.8	36.4	31.7	33.6	33.4	31.6	34.1	30.6	32.6
GSL-IA4	0.1	0.1	0	0	0	0.2	0.1	0.1	0	0	0.1	0	0	0	0.1	0.1	0.1	0.1	0.1
GSL-I B4	0.1	0	0	0	0	0.1	0.1	0.1	0	0	0	0	0	0	0	0.1	0	0	0

Each cell line at the indicated population doubling levels (PDLs) was applied for lectin microarray analysis

### Cell growth rate and morphological change of elderly-derived TIG-101 and TIG-102 cells

**Fig. 3:**
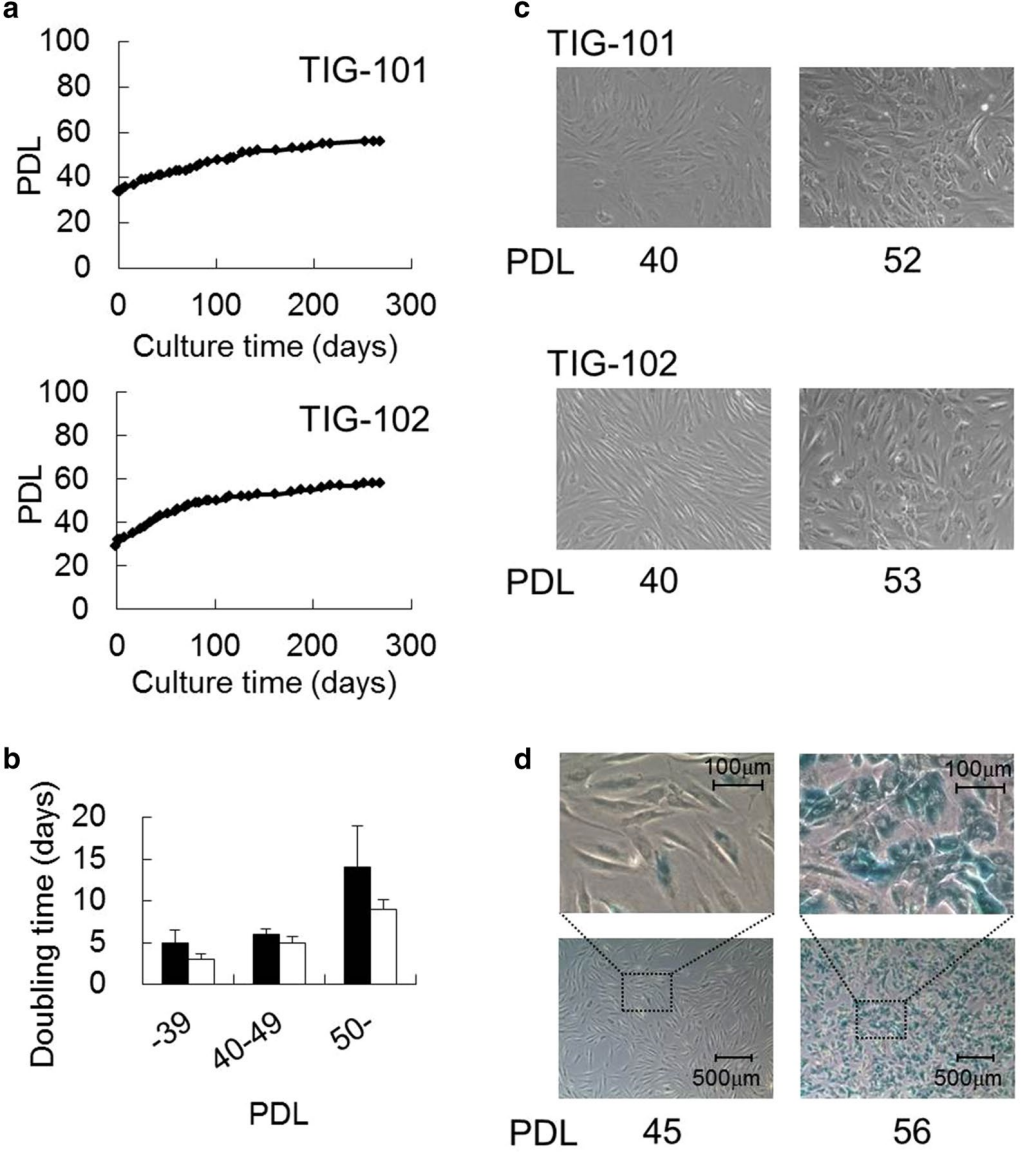
Growth curves and morphologies of human diploid fibroblasts derived from elderly (TIG-101 and TIG-102). **a** TIG-101 and TIG-102 proliferation rates were plotted as PDL for approximately 280-day culture (each n = 1). **b** *Bar graph* representation of the average doubling time of the cells at each PDL (mean + SE, TIG-101: n = 4–10; TIG-102: n = 5–9). *Closed* and *opened bars* represent TIG-101 and TIG-102 cell lines, respectively. **c** Cell shapes of TIG-101 and TIG-102 fibroblasts at PDLs 40 and 52, and PDLs 40 and 53, respectively. **d** TIG-102 cells at PDLs 45 and 56 were stained for SA-β-galactosidase. The* upper panels* are a magnification of the squared area in the* lower panels*

To compare the characteristics of fetal and elderly-derived adult cells, the growth rates of TIG-101 and TIG-102 cultures were observed. TIG-101 grew slowly, reaching approximately PDL 50 after 130-day culture, and TIG-102 reached approximately PDL 50 after 95-day culture (Fig. 3a). For both lines, the cells at approximately PDL 60 were in a state of growth arrest, suggesting cellular senescence. In fact, the average doubling time over PDL 50 (TIG-101: n = 5; TIG-102: n = 9) for both lines was about three times as long as that at PDLs 32–39 (Fig. 3b). In addition, although TIG-101 and TIG-102 exhibited spindle shapes at PDL 40, both cell types were flat after PDL 50 (Fig. 3c). TIG-102 was slightly stained with SA-β-galactosidase at PDL 45, but at late passage (>PDL 50) SA-β-galactosidase activity increased (Fig. 3d). However, although the growth of elderly-derived cell was slow, the early-passage cells (<PDL 40) were not senescence. These results suggested that senescence was substantively initiated for each elderly-derived cell line over PDL 50.

### Cellular senescence-dependent changes of cell surface glycans in TIG-101 and TIG-102 fibroblasts

**Fig. 4:**
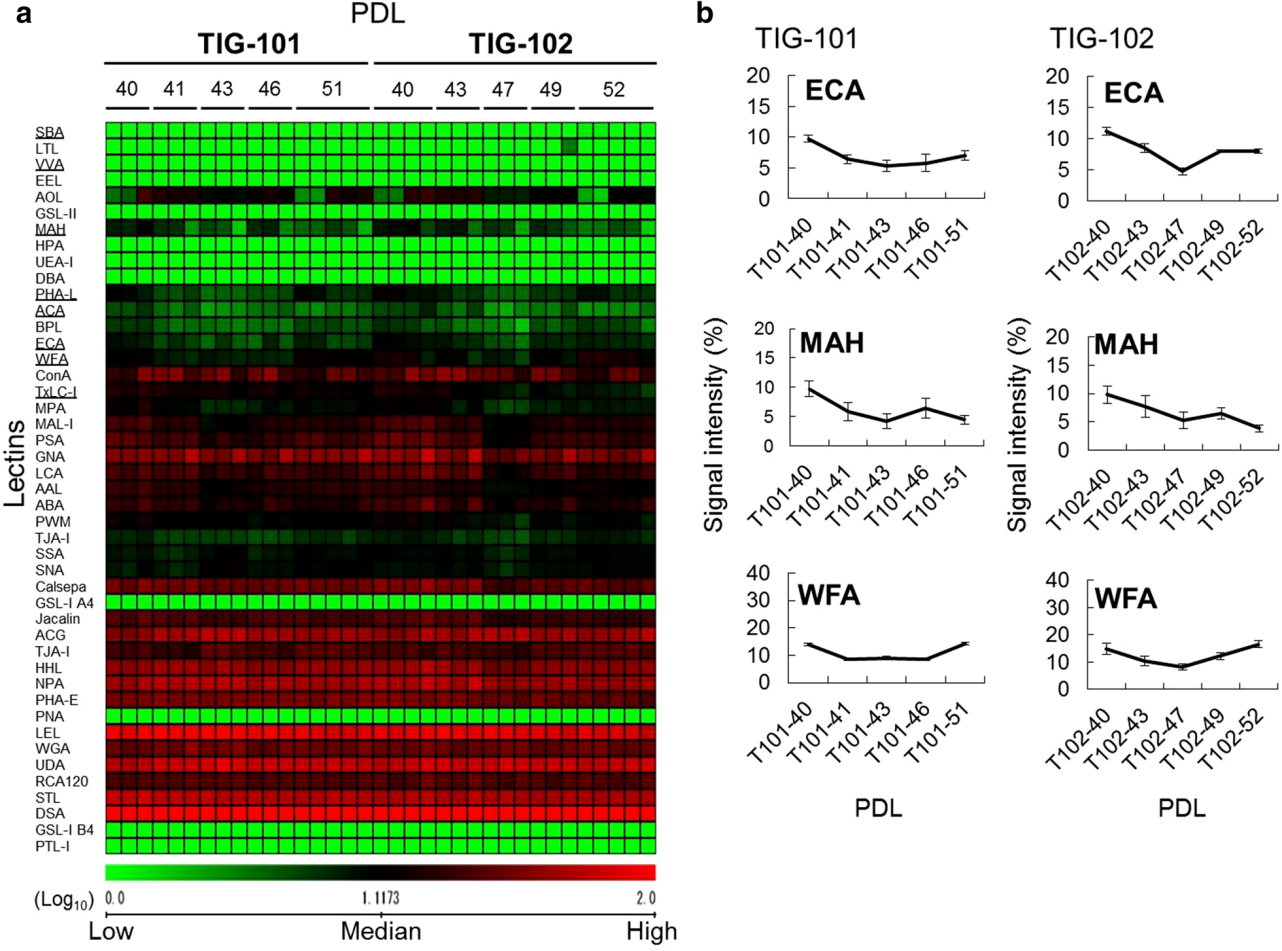
Lectin microarray analysis of cellular senescence in TIG-101 and TIG-102 at various PDLs. **a** Heat map representation of the (log_10_-transformed) lectin microarray data related to cellular senescence in the TIG-101 and TIG-102 cell lines. The *rows* represent lectins and the *columns* represent TIG-101 and TIG-102 cell lines at PDLs 40–51 and PDLs 40–52, respectively. The *color scale* indicates low (*green*) to high (*red*) signal intensity. **b** *Line graph* representation of the signal intensity (%) at each PDL for changed lectins. There are three representative patterns including ECA, MAH and WFA. The data are represented as the mean ± SE (n = 3–5)

To investigate the glycan profiles associated with each PDL in TIG-101 and TIG-102 cells, lectin microarray analyses were performed (Table 1). Figure 4a shows the heat map of lectin microarray signals for both lines. The signal intensities of *O*-glycan-binders such as SBA (GalNAc-binder) and VVA (GalNAc-binder), and those of large-antennary *N*-glycan-binders such as PHA-L and ECA, initially decreased and then slightly increased in both lines (Fig. 4b; Additional file 2: Figure S2; Table 1). The signal intensities of *N*-glycan-binders such as TxLC-I [Manα1-3(Manα1-6)Man- and GalNAc-binder], and those of *O*-glycan-binders such as ACA (Galβ1-3GalNAc-binder) and MAH (Siaα2-3Galβ1-3GalNAc-binder) decreased with passage. The signal intensity of WFA initially decreased and then increased slightly compared to early-passage levels. These results suggested that the large-antennary *N*-glycan form and *O*-glycans such as the Tn-antigen (GalNAc-Ser/Thr) were decreased; however their glycan profile changes were not greatly.

### Comparison of cell surface glycans between fetus- and elderly-derived cells

**Fig. 5:**
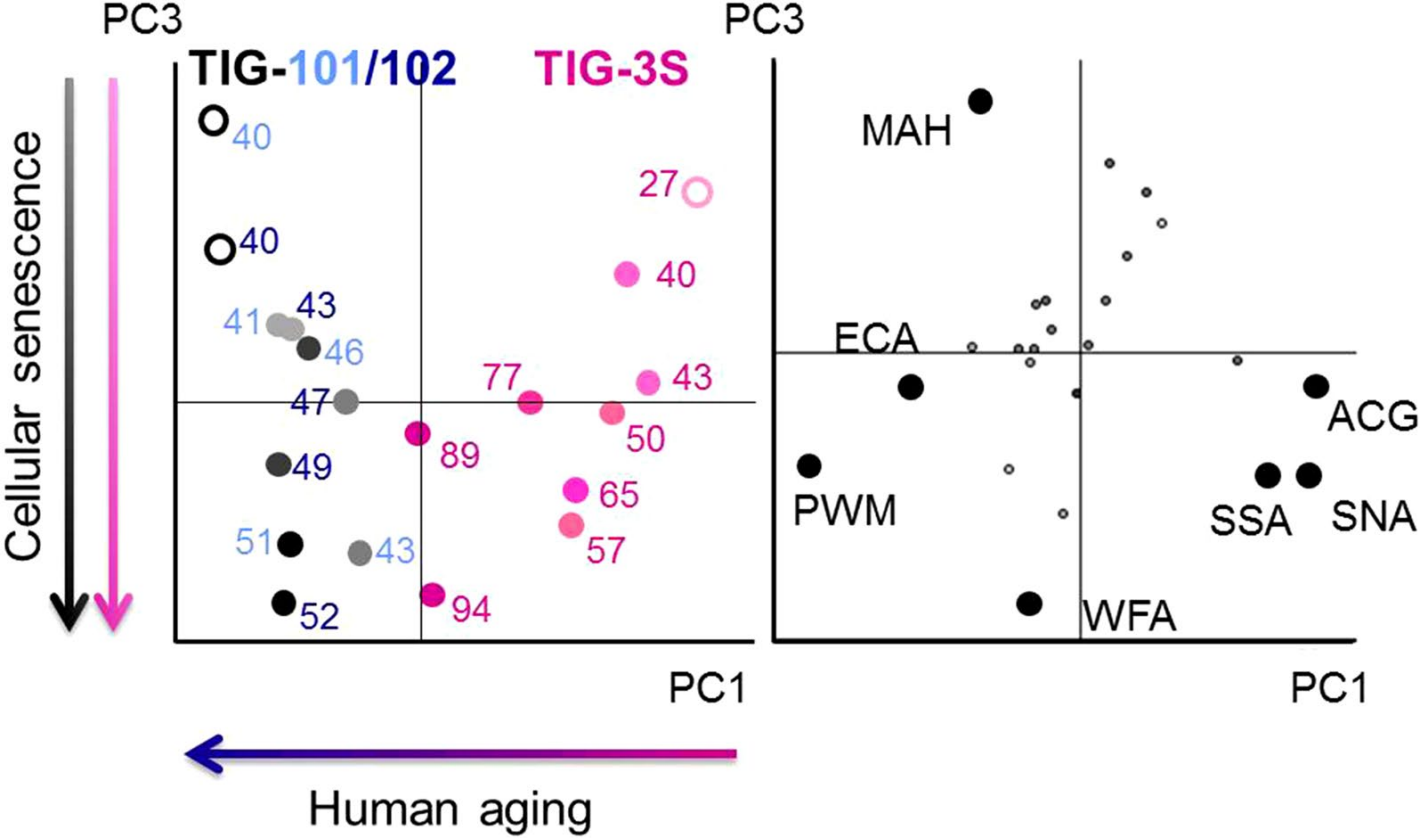
Biplot for PCA analysis. PC1 represents human aging and PC3 represents cellular senescence. The *pink*, *light blue*, and *dark blue* *labels* represent TIG-3S, TIG-101, and TIG-102 cell lines, respectively. Color gradients (light to dark) reflect cellular senescence (young to aged). *Closed circles* represent distinguishable lectins of human aging or cellular senescence. *Left panel* cell passage replications; *right panel* lectin replications shown as a biplot

To examine whether the changes of total glycan profiles during the cell passage process correlated with cell source age, the microarray data for TIG-3S, TIG-101, and TIG-102 were compared (Additional file 3: Figure S3). Hierarchical clustering analysis of the total glycan profiles revealed that TIG-3S and TIG-101/TIG-102 had individual glycan characters, whereas the glycan character of TIG-3S increasingly resembled those of TIG-101 and TIG-102 with increasing passage number (Additional file 4: Figure S4). Figure 5 presents the principal component analysis (PCA) results for 24 lectins in a biplot. PC3 appeared to correlate to cellular passage in all three lines. The positions of each PDL in the PC3 axis show the degree of passage-number, representing the gradual shift from young to aged cells. MAH plotted toward the positive direction and WFA plotted toward the negative direction of PC3. On the other hand, PC1 discriminated between TIG-3S and TIG-101/TIG-102, which plotted clearly toward the positive and the negative direction, respectively. ACG (Siaα2-3Galβ1-4GlcNAc-binder), SNA (Siaα2-6Gal-binders), and SSA (Siaα2-6Gal-binders) plotted toward the positive direction and PWM [(GlcNAc)_n_- and (Galβ1-4GlcNAc)_n_-binder] plotted toward the negative direction of PC1 as lectins differentiated these cells. Notably, aging TIG-3S PC1 localization approximated that of elderly-derived cells upon cellular senescence.

These results suggested that α2-3sialylation of the *O*-glycan form decreased with cellular senescence and α2-6sialylation of the *N*- and *O*-glycan forms and α2-3sialylation of the *N*-glycan form decreased with human aging.

## Discussion

Late-passage cells exhibit various phenomena associated with cellular senescence such as elevated SA-β-galactosidase activity, cell hypertrophy, and decreased proliferative capacity in vitro. *In vivo*, signs of human aging such as a decline of biological function appear with chronological age. Thus, the accumulation of cellular senescence appears to influence human aging. The increase in various diseases with aging is likely induced by its negative effects on biological function. However, it is not clear whether a correlation exists between cellular senescence and human aging. To better understand human aging to facilitate treatment and prevention of its effects, we investigated the glycan profile changes associated with human cellular senescence and aging.

In this report, we compared glycan profile characteristics and continuous glycan changes between fetus- (TIG-3S) and elderly-derived (TIG-101 and TIG-102) cells. When the growth potential of TIG-3S cells declined after PDL 80, the glycan profile was found to be significantly changed. The glycan profiles of both elderly-derived lines were similarly changed at late passage. For example, the MPA signal in TIG-3S increased and TxLC-I in TIG-101 and TIG-102 decreased with passage. This suggests that the cellular senescence process was related to the change in glycan composition of the cell surface. As the WFA signal in TIG-3S significantly increased at middle passage (>PDL 50) prior to the morphological changes of cellular expansion and growth arrest, we reason that the glycan changes occurred before the morphological changes. Furthermore, the small alterations observed in the lectin microarray data from elderly-derived lines were consistent with their slow growth rate. Considering that the alteration of MAH and WFA signals significantly attributed to PC1, it appears that the Galβ1-3GalNAc structure was covered with α2-3Sia residues in early-passage cells and that the amount of α2-3sialilated *O*-glycans decreased with cellular senescence. Because of a deletion of the α2-3 Sia residues on *O*-glycans, the Galβ1-3GalNAc structures on *O*-glycans exposure increased with cellular senescence. Despite the relatively limited number of cell lines used in this study, these data were in agreement with those of independent analysis we performed in other fibroblasts and various other cell types (unpublished data).

On the other hand, the overall signals of the α2-6 and α2-3sialylated *N*-glycans and *O*-glycans of fetus-derived cells were significantly stronger than those of elderly-derived cells, although the profiles tended to converge upon late passages. At that time, the glycan profile of the fetus-derived cells had greatly changed and resembled that of elderly-derived cells. It has been previously reported that fetal cell surface *N*-glycan α2-6 Sia residues decrease because of decreased ST6Gal I gene expression during cellular senescence [9]. Furthermore, extrinsic factor-induced rapid cellular senescence of adenocarcinoma cells leads to enhanced galactose residue cell surface exposure concomitant with increasing β1-4GalT [24]. Consequently, it has been suggested that desialylation impacts cellular senescence. Additionally, it has been shown that α2-3 and α2-6sialylation of *N*-glycans in adult tissue-derived cells of pregnant woman are altered during gestation and with age [25]. Functionally, the migration of human skin fibroblasts from elderly donors was found to be reduced and the migration was shown to differ between early- and late-passage cells by Kondo et al. in 1992 [24]. Thus, we speculate that the glycan changes of senescent cells are important for the mechanism of biological aging.

We note that the observed senescence-associated decreased sialylation and increased galactose exposure might be related to age-related disease as well as human aging. However, various sialylations have been often proposed as biomarkers for the genetic disease such as cancer [26]. This suggests that the mechanism of glycosylation differs between dysfunction with human aging, including age-related disease, and genetic disease. Therefore, we infer that desialylated senescent cells, which gradually accumulate in vivo, have detrimental effects on biological functions such as signal transduction and molecular recognition with human aging. To address these issues, quantitative analyses of detailed glycan changes associated with human aging and biological function will be required.

In addition to broadening our understanding of cellular function during aging in general, the establishment of a biomarker of cellular aging will facilitate the study of elderly patient-derived adult or stem cells, which are being used in various clinical trials [27–30]. It has been reported that stem cell aging is associated with the suppression of tissue regeneration and with malignant transformation [31, 32]. Disruption of these mechanisms possibly is an additional factor contributing to disease related to aging. Therefore, it is important to evaluate the potential efficacies of cells used as the source of regenerative therapy as well as to identify the optimal cells for such usage. Accordingly, knowledge of the glycan modifications present on aging cells will be useful in the identification of appropriate therapeutic cells.

## Methods

### Cell culture

The fetus-derived TIG-3S, 86-year-old subject-derived TIG-101, and 97-year-old subject-derived TIG-102 fibroblast cell lines were purchased from the Health Science Research Resources Bank (Osaka, Japan); the respective PDLs were 23, 34, and 29. Cell proliferative capacity was assessed by calculating the total number of PDLs using the formula PDL = log_2_(total number of cells/initial number of cells). Here, the PDL counts were rounded up after the decimal point. Cells were maintained in Dulbecco’s modified Eagle medium (Wako Pure Chemical Industries, Osaka, Japan) containing 10 % fetal bovine serum (Cell Culture Technologies, Gravesano, Switzerland) supplemented with 50 U/ml penicillin and 50 μg/ml streptomycin (Gibco, Grand Island, NY, USA). All cultures were subcultivated in 100 mm plastic dishes (Falcon, San Jose, CA, USA) at 37 °C under humidified 5 % CO_2_. When the cultures reached confluence at 3–4 days (TIG-3S) or 1–2 weeks (TIG-101 and TIG-102) of subcultivation, the cells were removed from the dish by treatment with 0.25 % trypsin–EDTA solution (IBL, Gunma, Japan) and subcultivated further using 0.4 to 0.5 × 10^6^ cells. However, later passage cultures of TIG-101 and TIG-102 were subcultivated using 0.1 to 0.3 × 10^6^ cells. The doubling time was calculated as the time in culture required for each PDL (days/PDL). All cell pellets were collected for assessment according to the PDLs shown in Table 2. For comparison of cell surface glycan profiles, cell pellets were subjected to lectin microarray analysis.

**Table 2:** Population doubling levels (PDLs) applied for assessments

Cell line	PDL									
TIG-3S	27	40	43	50	57	65	77	83^b^	89	94
TIG-101	40	41	43	46	51	52^a^				
TIG-102	40	43	45^b^	47	49	52	53^a^	56^b^		

Cells were applied for morphological and histochemical analysis

^a^Only morphological observation

^b^Only histochemical stain

### Senescence-associated β-galactosidase (SA-β-galactosidase) detection

SA-β-galactosidase activity in cultured cells was histochemically detected using the Senescence Detection Kit (Calbiochem, EMD Biosciences, Darmstadt, Germany). In brief, the culture medium was removed and the cultured cells were rinsed with 2 ml of phosphate-buffered saline (PBS) and then fixed with 1 ml of fixative solution at room temperature for 15 min. After rinsing with PBS, the cells were stained with 1 ml of staining solution mixture (staining solution: staining supplement: 20 mg/ml X-gal, 94:1:5) at 37 °C for 17 h. After incubation, the stained cells were observed under a microscope.

### Lectin microarray analysis

Protein extracts of TIG-3S, TIG-101, and TIG-102 cell pellets (approximately 5 × 10^4^ to 1 × 10^6^ cells) collected at various PDLs were isolated as hydrophobic protein fractions using a CelLytic MEM Protein Extraction kit (Sigma, St. Louis, MO, USA) as described previously [23, 33]. Total proteins including glycoproteins (200 ng) were labeled with Cy3 mono-reactive dye (GE Healthcare, Buckinghamshire, UK) in PBS containing 0.5 % Triton X-100 at room temperature for 1 h. To remove excess Cy3 mono-reactive dye, the reaction solution was diluted with 20 μl of probing buffer (Tris-buffered saline containing 1 % Triton X-100, 1 mM CaCl_2_, and 1 mM MnCl_2_, pH 7.4), and applied to a spin-type desalting column loaded with Sephadex G-25 fine matrix (GE Healthcare). The Cy3-labeled glycoprotein solution (60 µl) was applied to a LecChip (Glyco Technica, Yokohama, Japan). After incubation at 4 °C for approximately 17 h, the reaction solution was discarded. The glass slide was washed three times with probing buffer before the LecChip was scanned using the evanescent-field fluorescence scanner GlycoStation™ Reader 1200 (Glyco Technica). Each sample was measured three to five times independently. All data were analyzed using GlycoStation™ Tools Signal Capture 1.0 and GlycoStation™ Tools Pro 1.0 (Glyco Technica). To expand the dynamic range, the data were subjected to a gain-merging procedure, and the merged data were normalized using max-normalization as described previously [19].

### Statistical analysis

The lectin microarray data was analyzed using hierarchical clustering and PCA by means of pair-wise comparison, using http://www.lgsun.grc.nia.nih.gov/ANOVA/ (false discovery rate <0.05). The data was also analyzed and displayed using TIGR MultiExperiment Viewer (http://www.tm4.org/mev.html). The mean value of the lectin microarray data was used for each respective PCA.

### Additional files

**Additional file 1: Figure S1.** Lectin microarray analysis of cellular senescence in TIG-3S fibroblasts at various PDLs. *Line graph* representation of signal intensity (%) at each PDL in selected significantly changed lectins. The signal intensities of MAL-I, Calsepa as well as MPA, changed at late passage. The signal intensities of TJA-II, ECA, PHA-L as well as BPL, changed during long passage. The data are represented as the mean ± SE (n = 3). Heat map plots are shown in each *line graph* with the *color scale * indicating low (*blue*) to high (*yellow*) signal intensity.

**Additional file 2: Figure S2.** Lectin microarray analysis of cellular senescence in TIG-101 and TIG-102 fibroblasts at various PDLs. *Line graph* representation of signal intensity (%) at each PDL in selected changed lectins. The signal intensities of SBA, VVA, PHA-L as well as ECA, first decreased and then slightly increased. The signal intensities of TxLC-I, ACA as well as MAH, decreased gradually. The data are represented as the mean ± SE (n = 3–5). Heat map plots are shown in each *line graph *with the *color scale *indicating low (*blue*) to high (*yellow*) signal intensity.

**Additional file 3: Figure S3.** Lectin microarray analysis of TIG-3S, TIG-101, and TIG-102 cell lines at various PDLs. Heat map representation of the (log_10_-transformed) lectin microarray data. The *rows* represent the lectins and the *columns* represent TIG-3S, TIG-101, and TIG-102 cell lines at PDLs 27–94, PDLs 40–51 and PDLs 40–52, respectively. The *color scale *indicates low (*green*) to high (*red*) signal intensity.

**Additional file 4: Figure S4.** Hierarchical clustering of glycan profile for TIG-3S (*pink*), TIG-101 (*light blue*), and TIG-102 (*dark blue*). The lectin microarray data were analyzed at PDLs 27–94, PDLs 40–51, and PDLs 40–52, respectively.

## Abbreviations

Fuc: fucose
Gal: galactose
GalNAc: *N*-acetylgalactosamine
Glc: glucose
GlcNAc: *N*-acetylglucosamine
Man: mannose
PBS: phosphate-buffered saline
PCA: principal component analysis
PDL: population doubling level
SA-β-galactosidase: senescence-associated β-galactosidase
Sia: sialic acid
