# Supplementary figures and images for: N- and O-glycan cell surface protein modifications associated with cellular senescence and human aging
Source: Cell Biosci. 2016 Feb 18;6:14. doi: 10.1186/s13578-016-0079-5 (PMC4757982; doi:10.1186/s13578-016-0079-5)

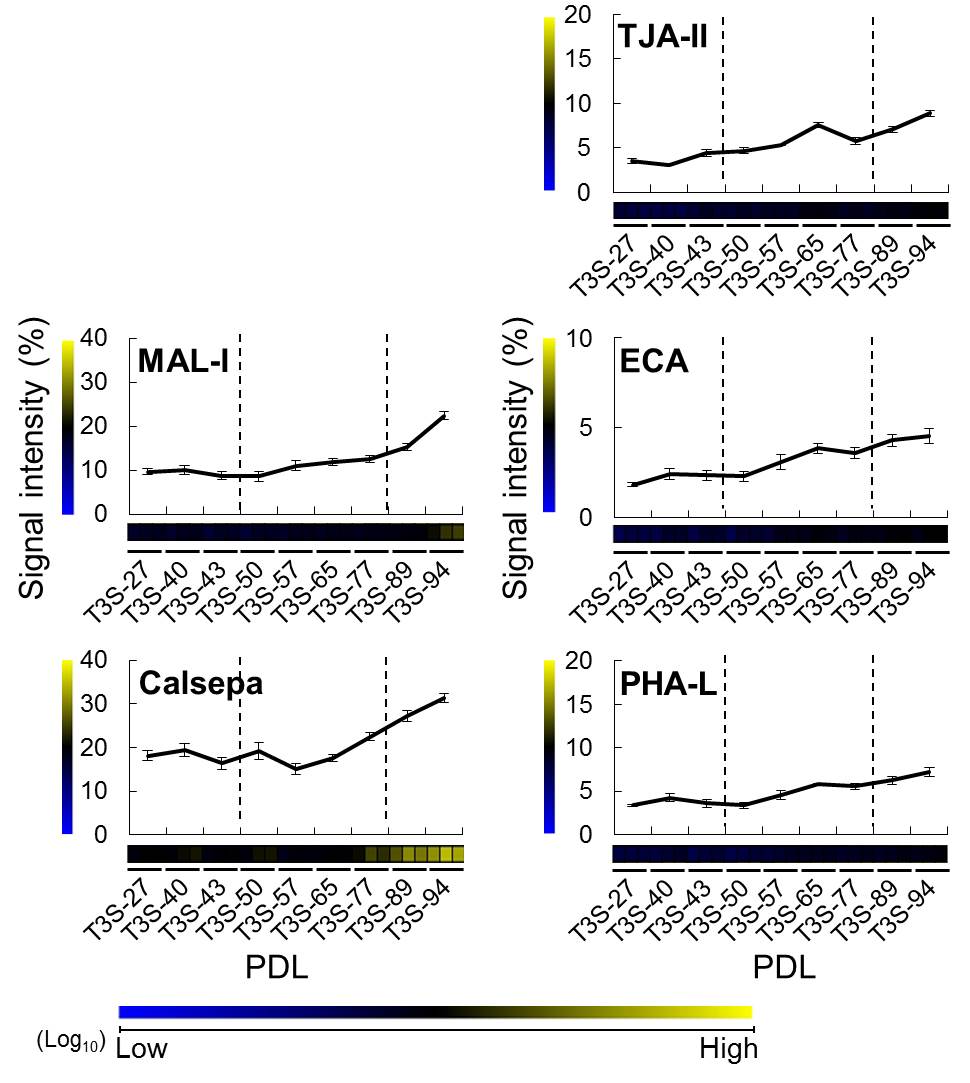

Supplement: Supplementary file 1 — Additional file 1: Figure S1. Lectin microarray analysis of cellular senescence in TIG-3S fibroblasts at various PDLs. Line graph representation of signal intensity (%) at each PDL in selected significantly changed lectins. The signal intensities of MAL-I, Calsepa as well as MPA, changed at late passage. The signal intensities of TJA-II, ECA, PHA-L as well as BPL, changed during long passage. The data are represented as the mean ± SE (n = 3). Heat map plots are shown in each line graph with the color scale indicating low (blue) to high (yellow) signal intensity. [file 13578_2016_79_MOESM1_ESM.jpg]

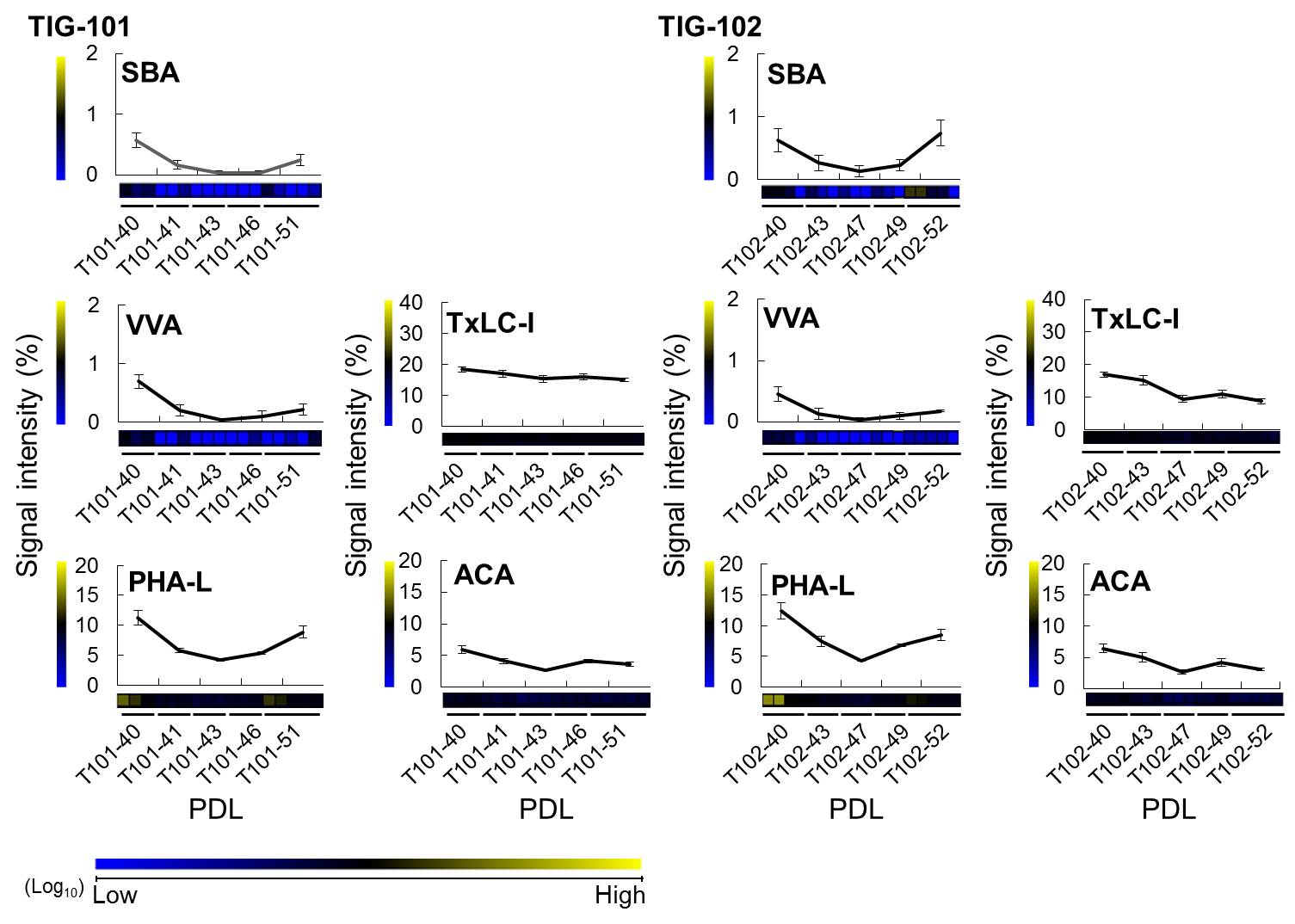

Supplement: Supplementary file 2 — Additional file 2: Figure S2. Lectin microarray analysis of cellular senescence in TIG-101 and TIG-102 fibroblasts at various PDLs. Line graph representation of signal intensity (%) at each PDL in selected changed lectins. The signal intensities of SBA, VVA, PHA-L as well as ECA, first decreased and then slightly increased. The signal intensities of TxLC-I, ACA as well as MAH, decreased gradually. The data are represented as the mean ± SE (n = 3–5). Heat map plots are shown in each line graph with the color scale indicating low (blue) to high (yellow) signal intensity. [file 13578_2016_79_MOESM2_ESM.jpg]

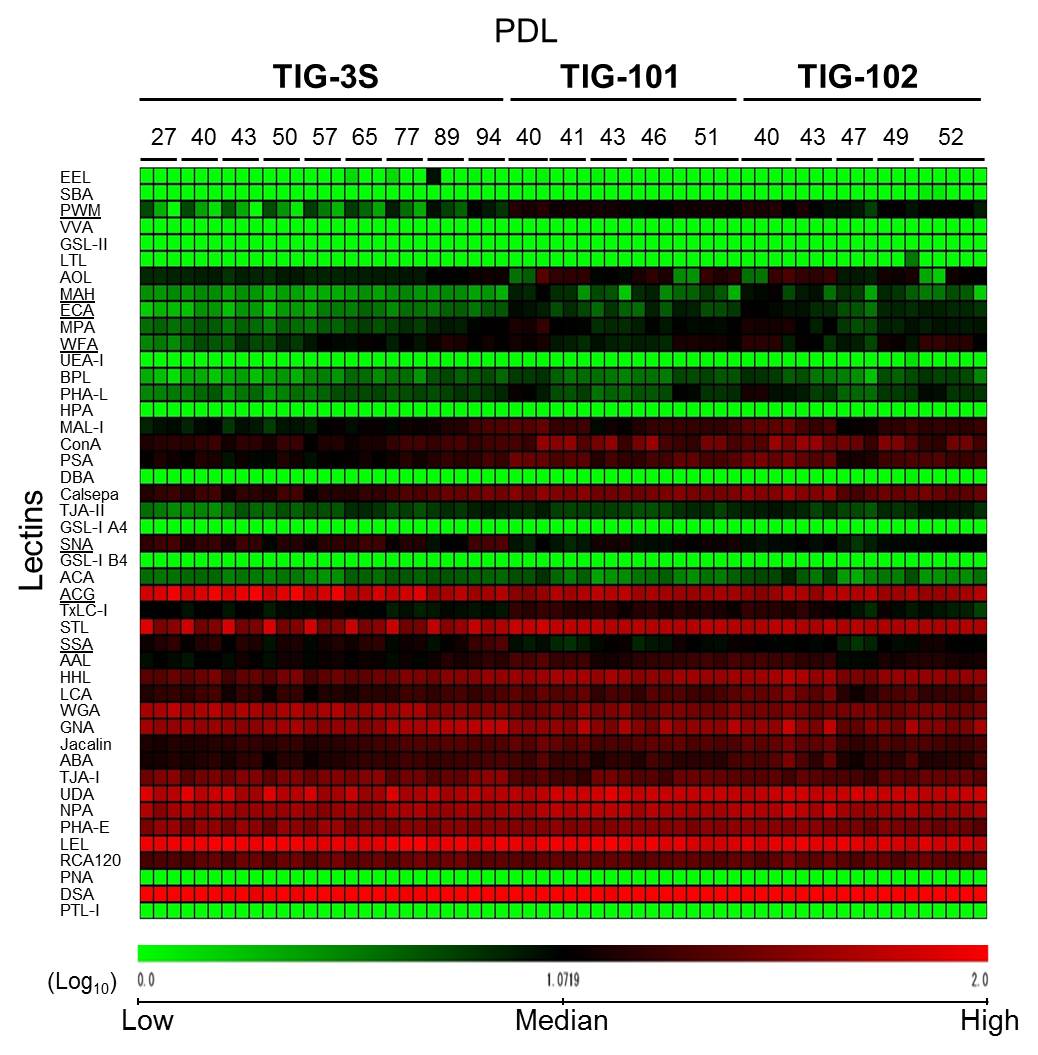

Supplement: Supplementary file 3 — Additional file 3: Figure S3. Lectin microarray analysis of TIG-3S, TIG-101, and TIG-102 cell lines at various PDLs. Heat map representation of the (log10-transformed) lectin microarray data. The rows represent the lectins and the columns represent TIG-3S, TIG-101, and TIG-102 cell lines at PDLs 27–94, PDLs 40–51 and PDLs 40–52, respectively. The color scale indicates low (green) to high (red) signal intensity. [file 13578_2016_79_MOESM3_ESM.jpg]

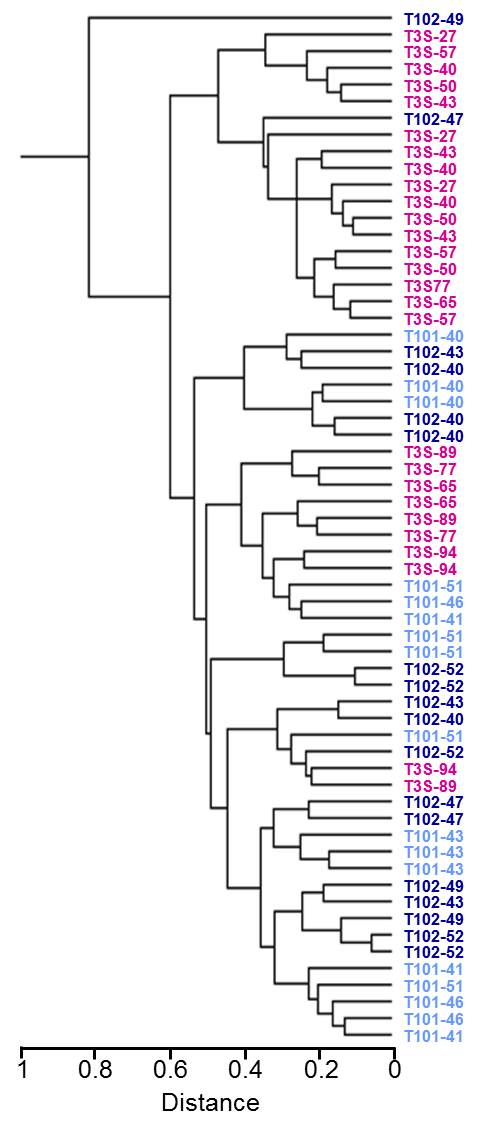

Supplement: Supplementary file 4 — Additional file 4: Figure S4. Hierarchical clustering of glycan profile for TIG-3S (pink), TIG-101 (light blue), and TIG-102 (dark blue). The lectin microarray data were analyzed at PDLs 27–94, PDLs 40–51, and PDLs 40–52, respectively. [file 13578_2016_79_MOESM4_ESM.jpg]
